# Supplementary material for: Systematic Review and Meta-Analysis of microRNA-7-5p Expression and Biological Significance in Head and Neck Squamous Cell Carcinoma
Source: Cancers (Basel). 2025 Oct 4;17(19):3232. doi: 10.3390/cancers17193232 (PMC12524308; doi:10.3390/cancers17193232)
Supplement: Supplementary file 1 [file cancers-17-03232-s001.zip › cancers-3795713-supplementary.pdf]

Table S1. PRISMA 2020 Checklist for reporting a systematic review and meta-analysis of individual participant data.

PRISMA-IPD Section/topic	Item No	Checklist item	Reported on page
**Title**			
Title	1	Identify the report as a systematic review and meta-analysis of individual participant data.	1
**Abstract**			
Structured summary	2	Provide a structured summary including as applicable:	1-2
		**Background**: state research question and main objectives, with information on participants, interventions, comparators and outcomes.	
		**Methods**: report eligibility criteria; data sources including dates of last bibliographic search or elicitation, noting that IPD were sought; methods of assessing risk of bias.	
		**Results**: provide number and type of studies and participants identified and number (%) obtained; summary effect estimates for main outcomes (benefits and harms) with confidence intervals and measures of statistical heterogeneity. Describe the direction and size of summary effects in terms meaningful to those who would put findings into practice.	
		**Discussion:** state main strengths and limitations of the evidence, general interpretation of the results and any important implications.	
		**Other:** report primary funding source, registration number and registry name for the systematic review and IPD meta-analysis.	
**Introduction**			
Rationale	3	Describe the rationale for the review in the context of what is already known.	2-3
Objectives	4	Provide an explicit statement of the questions being addressed with reference, as applicable, to participants, interventions, comparisons, outcomes and study design (PICOS). Include any hypotheses that relate to particular types of participant-level subgroups.	3
**Methods**			
Protocol and registration	5	Indicate if a protocol exists and where it can be accessed. If available, provide registration information including registration number and registry name. Provide publication details, if applicable.	3

Eligibility criteria	6	Specify inclusion and exclusion criteria including those relating to participants, interventions, comparisons, outcomes, study design and characteristics (e.g. years when conducted, required minimum follow-up). Note whether these were applied at the study or individual level i.e. whether eligible participants were included (and ineligible participants excluded) from a study that included a wider population than specified by the review inclusion criteria. The rationale for criteria should be stated.	3-4
Identifying studies - information sources	7	Describe all methods of identifying published and unpublished studies including, as applicable: which bibliographic databases were searched with dates of coverage; details of any hand searching including of conference proceedings; use of study registers and agency or company databases; contact with the original research team and experts in the field; open adverts and surveys. Give the date of last search or elicitation.	3-4
Identifying studies - search	8	Present the full electronic search strategy for at least one database, including any limits used, such that it could be repeated.	3-4
Study selection processes	9	State the process for determining which studies were eligible for inclusion.	3-4. 7
Data collection processes	10	Describe how IPD were requested, collected and managed, including any processes for querying and confirming data with investigators. If IPD were not sought from any eligible study, the reason for this should be stated (for each such study).	3-5, 7
		If applicable, describe how any studies for which IPD were not available were dealt with. This should include whether, how and what aggregate data were sought or extracted from study reports and publications (such as extracting data independently in duplicate) and any processes for obtaining and confirming these data with investigators.	
Data items	11	Describe how the information and variables to be collected were chosen. List and define all study level and participant level data that were sought, including baseline and follow-up information. If applicable, describe methods of standardising or translating variables within the IPD datasets to ensure common scales or measurements across studies.	3-5
IPD integrity	A1	Describe what aspects of IPD were subject to data checking (such as sequence generation, data consistency and completeness, baseline imbalance) and how this was done.	4-5
Risk of bias assessment in individual studies.	12	Describe methods used to assess risk of bias in the individual studies and whether this was applied separately for each outcome. If applicable, describe how findings of IPD checking were used to inform the assessment. Report if and how risk of bias assessment was used in any data synthesis.	6

Specification of outcomes and effect measures	13	State all treatment comparisons of interests. State all outcomes addressed and define them in detail. State whether they were pre-specified for the review and, if applicable, whether they were primary/main or secondary/additional outcomes. Give the principal measures of effect (such as risk ratio, hazard ratio, difference in means) used for each outcome.	6, Table S3-4
Synthesis methods	14	Describe the meta-analysis methods used to synthesise IPD. Specify any statistical methods and models used. Issues should include (but are not restricted to):<br>• Use of a one-stage or two-stage approach.<br>• How effect estimates were generated separately within each study and combined across studies (where applicable).<br>• Specification of one-stage models (where applicable) including how clustering of patients within studies was accounted for.<br>• Use of fixed or random effects models and any other model assumptions, such as proportional hazards.<br>• How (summary) survival curves were generated (where applicable).<br>• Methods for quantifying statistical heterogeneity (such as I^2 and τ^2).<br>• How studies providing IPD and not providing IPD were analysed together (where applicable).<br>• How missing data within the IPD were dealt with (where applicable).	6
Exploration of variation in effects	A2	If applicable, describe any methods used to explore variation in effects by study or participant level characteristics (such as estimation of interactions between effect and covariates). State all participant-level characteristics that were analysed as potential effect modifiers, and whether these were pre-specified.	6, 14
Risk of bias across studies	15	Specify any assessment of risk of bias relating to the accumulated body of evidence, including any pertaining to not obtaining IPD for particular studies, outcomes or other variables.	6, 24
Additional analyses	16	Describe methods of any additional analyses, including sensitivity analyses. State which of these were pre-specified.	3-5, 9
**Results**			
Study selection and IPD obtained	17	Give numbers of studies screened, assessed for eligibility, and included in the systematic review with reasons for exclusions at each stage. Indicate the number of studies and participants for which IPD were sought and for which IPD were obtained. For those studies where IPD were not available, give the numbers of studies and participants for which aggregate data were available. Report reasons for non-availability of IPD. Include a flow diagram.	7
Study characteristics	18	For each study, present information on key study and participant characteristics (such as description of interventions, numbers of participants, demographic data, unavailability of outcomes, funding source, and if applicable duration of	Table S3-4

		follow-up). Provide (main) citations for each study. Where applicable, also report similar study characteristics for any studies not providing IPD.	
IPD integrity	A3	Report any important issues identified in checking IPD or state that there were none.	3-4, Table S2
Risk of bias within studies	19	Present data on risk of bias assessments. If applicable, describe whether data checking led to the up-weighting or down-weighting of these assessments. Consider how any potential bias impacts on the robustness of meta-analysis conclusions.	4, 24
Results of individual studies	20	For each comparison and for each main outcome (benefit or harm), for each individual study report the number of eligible participants for which data were obtained and show simple summary data for each intervention group (including, where applicable, the number of events), effect estimates and confidence intervals. These may be tabulated or included on a forest plot.	8, 9, 11, Table S3-4
Results of syntheses	21	Present summary effects for each meta-analysis undertaken, including confidence intervals and measures of statistical heterogeneity. State whether the analysis was pre-specified, and report the numbers of studies and participants and, where applicable, the number of events on which it is based.	Figures 2-4
		When exploring variation in effects due to patient or study characteristics, present summary interaction estimates for each characteristic examined, including confidence intervals and measures of statistical heterogeneity. State whether the analysis was pre-specified. State whether any interaction is consistent across trials.	
		Provide a description of the direction and size of effect in terms meaningful to those who would put findings into practice.	
Risk of bias across studies	22	Present results of any assessment of risk of bias relating to the accumulated body of evidence, including any pertaining to the availability and representativeness of available studies, outcomes or other variables.	Figure 3
Additional analyses	23	Give results of any additional analyses (e.g. sensitivity analyses). If applicable, this should also include any analyses that incorporate aggregate data for studies that do not have IPD. If applicable, summarise the main meta-analysis results following the inclusion or exclusion of studies for which IPD were not available.	9-10
**Discussion**			
Summary of evidence	24	Summarise the main findings, including the strength of evidence for each main outcome.	22
Strengths and limitations	25	Discuss any important strengths and limitations of the evidence including the benefits of access to IPD and any limitations arising from IPD that were not available.	24-25

Conclusions	26	Provide a general interpretation of the findings in the context of other evidence.	22-26
Implications	A4	Consider relevance to key groups (such as policy makers, service providers and service users). Consider implications for future research.	22-26
**Funding**			
Funding	27	Describe sources of funding and other support (such as supply of IPD), and the role in the systematic review of those providing such support.	25-26

© Reproduced with permission of the PRISMA IPD Group, which encourages sharing and reuse for non-commercial purposes

Table S2. Characteristics of studies with reported fold changes only.

Article ID	Country	Tissue	Isolation Method	Detection Method	Tumour Site	Normal Site	Sex	Age	Risk Factors	Stage	Normalisation Method	Tumour (N)	Normal (N)	Fold change	p
Chattopadhyay, 2016	India	Frozen	AllPrep DNA/RNA Mini Kit (Qiagen)	RT-qPCR (Taqman)	Oral cavity (100%)	Normal oral cavity tissue	Male (53%), Female (47%)	57 (30-77)	Tobacco (91%)	NR	$2^{\Delta\Delta Ct}$ (RNU44	23	20	2	<0.05
De Sarkar, 2014	India	Frozen	mirVana RNA Isolation Kit (Ambion)	TLDA Human miR Panel (Applied Biosystems)/RT-qPCR (Taqman)	Buccal mucosa (33%), cheek (39%), gingiva (17%), retro-molar region (6%), buccal vestibule (6%)	Matched adjacent normal tissue	Male (56%), Female (44%)	51 (39-80)	Tobacco (100%)	I (22%), II (17%), III (56%), IV (6%)	Geomean (RNU-44, RNU-48 and mmu-6)	18	18	3.89	0.00048
Ganci, 2013	Italy	RNAlater	miRNeasy kit (Qiagen)	Human miRNA Microarray (V2) 8x15K/SurePrint G3 Human v16 miRNA 8x60K	Oral cavity (63%), larynx (19%), hypopharynx (10%), oropharynx (9%)	Matched adjacent normal tissue	Male (77%), Female (23%)	62 (19-86)	Tobacco (81%), Alcohol (67%), HPV+ (3%), p53 mut (100%)	NR	Percentile (25th-75th) normalised and log2-transformed	70	66	3.98	$1.55x10^{-10}$
Ganci, 2017	Italy	RNAlater	miRNeasy kit (Qiagen)	Human miRNA Microarray (V2) 8x15K/SurePrint G3 Human v16 miRNA 8x60K	Oral cavity (60%), larynx (25%), hypopharynx (7%), oropharynx (8%)	Matched adjacent normal tissue	Male (70%), Female (29%)	62	Tobacco (64%), Alcohol (50%), HPV+ (4%)	NR	Background subtracted, quantile normalised and log2-transformed	66	66	4.21	$1.23x10^{-15}$
Ganci, 2016	Italy	RNAlater	miRNeasy kit (Qiagen)	Human miRNA Microarray (V2) 8x15K/SurePrint G3 Human v16 miRNA 8x60K	Oral cavity (100%)	Matched adjacent normal tissue	Male (62%), Female (38%)	64	Tobacco (71%), Alcohol (58%), HPV+ (1%), p53 mut (60%)	NR	Percentile (25th-75th) normalised and log2-transformed	74	38	1.9	<0.001
Gao, 2020	China	NR	Trizol Reagent (Invitrogen)	Illumina HiSeq 2000	Larynx glottis (51%), supraglottis (39%), subglottis	Matched adjacent	Male (94%),	NR	NR	I (27%), II (22%),	NR	107	107	2.87	$3.36x10^{-34}$

					(3%), transglottis (7%)	normal mucosa	Female (6%)			III (22%), IV (28%)					
Kikkawa, 2010	Japan	Fresh frozen	Trizol Reagent (Invitrogen)	TLDA Human miR Panel (Applied Biosystems)	Hypopharynx (100%)	Matched adjacent normal tissue	Male (80%), Female (20%)	60 (52-74)	HPV+ (0%)	I (10%), III (10%), IV (80%)	Global and endogenous gene normalisation (RNU-44 and RNU-48)	10	10	9.3	0.00018
Koshizuka, 2017	Japan	NR	Trizol Reagent (Invitrogen)	Illumina Genome Analyzer IIx (Homo sapiens)	Hypopharynx (67%), larynx (33%)	Matched adjacent tissue	Male (100%)	66 (45-82)	NR	IV (100%)	NR	6	6	3.41	0.0596
Ramdas, 2009	America	Fresh frozen	Trizol Reagent (Invitrogen)	mirVana miRNA Bioarray Microarray (Ambion)	Tongue (60%), laryngopharynx (20%), floor of mouth (20%)	Matched adjacent normal tissue	NR	NR	NR	NR	Background subtracted, global median normalised, log2-transformed	5	5	10.03	0.00742
Schneider, 2018	Poland	Fresh frozen	Qiazol Reagent (Qiagen)	Illumina HiSeq 2500	Tongue (40%), floor of mouth (60%)	Matched adjacent normal tissue	Male (40%), Female (60%)	64 (58-70)	HPV+ (0%)	III (100%)	NR	5	5	19.87	0.0004
Zhou, 2017	America	NA	RNeasy kit (Qiagen)	microRNA Ready-to-Use PCR, Human panel I (Exiqon)	Tongue (50%), gingiva (40%), floor of mouth (5%), buccal mucosa (5%)	Unpaired normal brush biopsy	Male (60%), Female (40%)	62 (37-90)	Tobacco/Betel nut (40%)	NR	Normalised to reference sample	20	17	6	0.000041

[1] NR, Not Reported

Table S3. Newcastle-Ottawa Scale (NOS) to assess quality of included studies.

	**Selection**				**Comparability**		**Exposure**			
**Study**	**Adequate case definition**	**Representative-ness of cases**	**Selection of controls**	**Definition of controls**	**On the basis of decision**	**On the basis of analysis**	**Ascertainment of exposure**	**Same method of ascertainment for cases and control**	**Non-response rate**	**Total Quality Score**
Avissar 2009	★	★				★	★	★	★	6
Bruce 2015	★		★			★	★	★	★	6
Cervigne 2009	★		★	★	★	★	★	★	★	8
Chamorro 2018	★		★	★		★	★	★	★	7
Fukumoto 2014	★		★	★	★	★	★	★	★	8
Jung 2012	★					★	★	★	★	5
Lapa 2019	★		★			★	★	★	★	6
Li 2011	★		★			★	★	★	★	6
Liu 2012	★		★			★	★	★	★	6
Lyu 2014			★			★	★	★	★	5
Peng 2014b	★		★	★	★	★	★	★	★	8
Saito 2013			★		★	★	★	★	★	6
Severino 2013	★		★			★	★	★	★	6

Shi 2015[1]			★				★	★	★	4
Shiah 2014	★		★	★	★	★	★	★	★	8
Stansfield 2016	★	★	★	★	★	★	★	★	★	9
Xiao 2012			★			★	★	★	★	5
Yang 2018	★		★	★	★	★	★	★	★	8
Zhao 2019			★		★	★	★	★	★	6
Zhuang 2020	★	★	★	★	★	★	★	★	★	9
Lyu 2020	★		★	★	★	★	★	★	★	8
Wu 2020	★		★	★	★	★	★	★	★	8
Zheng 2018	★		★		★	★	★	★	★	7
MacLellan 2012			★		★	★	★	★	★	6
Shi 2019	★		★	★	★	★	★	★	★	8
Chattopadhyay 2016[2]				★		★	★	★	★	5
De Sarkar 2014	★		★	★	★	★	★	★	★	8
Ganci 2013	★	★	★	★	★	★	★	★	★	9
Ganci 2014			★	★	★	★	★	★	★	7
Ganci 2017	★	★	★	★	★	★	★	★	★	9
Gao 2020	★	★	★	★	★	★	★	★	★	9
Kikkawa 2010			★	★	★	★	★	★	★	7
Koshizuka 2017		★	★	★	★	★	★	★	★	8
Ramdas 2009			★	★	★	★	★	★	★	7
Schneider 2018			★	★	★	★	★	★	★	7
Zhou 2017	★		★	★	★	★	★	★	★	8

[1] 2 samples of matched tissues that were poorly annotated.

[2] All normal samples from unmatched individuals (all non-smokers) and no adjustment for confounders, no clinical information for cases.

Table S4. Characteristics of studies included in the meta-analysis with individual patient data.

Article ID	Accession number	Country	Tissue	Isolation Method	Detection Method	Tumour Site	Normal Site	Sex	Age	Risk Factors	Stage	Reported Outcomes	Normalisation Method	Tumour (N)	Normal (N)
Avissar, 2009	GSE11163	America	Fresh frozen	mirVana RNA Isolation Kit (Ambion)	mirVANA microRNA Bioarray (v2, Ambion)	Tongue, larynx, uvula, mouth	Tongue, larynx, uvula	NR	NR	HPV+ (19%)	NR	NR	Variance-stabilising transformation	16	5
Bruce, 2015	GSE70970	Canada	FFPE	Recover All Total Nucleic Acid Isolation Kit for FFPE (Ambion)	nCounter Human miRNA Assay (v1.0, Nanostring)	Nasopharynx (100%)	Nasopharynx	Male (71%), Female (29%)	51 (14-89)	NR	I (9%), II (23%), III (32%), IV (35%)	OS, DFS, Local/ Nodal/ Distant RFS	Variance-stabilising transformation	246	17

Cervigne, 2009	Supplementary Table 4	Canada	FFPE	Recover All Total Nucleic Acid Isolation Kit for FFPE (Ambion)	TLDA Human miR Panel (Applied Biosystems)	Oral cavity (100%)	Oral mucosa	NR	NR	NR	NR	NR	Log2-transformed and relative to normal tissue	16	11
Chamorro, 2018	GSE98463	Spain	Fresh frozen	mirVana Isolation Kit (Ambion)	Multispecies miRNA-4 Array (v4.1, Affymetrix)	Oral mucosa (69%), tongue (19%), mouth (6%),	Oral mucosa	Male (56%), Female (44%)	54 (18-75)	Tobacco (31%)	II (25%) IV (75%)	NR	Robust Multichip Average	8	8
Citron, 2017	GSE89000	Italy	Fresh frozen	Trizol (Invitrogen)	nCounter Human miRNA Assay (v1.0, Nanostring)	Hypopharynx (7%), larynx (11%),	None	Male (80%), Female (20%)	59 (43-82)	HPV+ (57%), p53 mutant (80%)	I (25%), II (25%), III (34%), IV (14%)	2-year Local RFS status	Normalised to internal positive spike control and housekeeping genes	44	0
De Cecco, 2017	GSE92595	Italy	FFPE	miRNeasy FFPE kit (Qiagen)	SurePrint G3 Human miRNA 8×60K microarray (Agilent)	Oral cavity (48%), oropharynx (23%), hypopharynx (10%), larynx (20%)	None	Male (78%), Female (23%)	62 (36-75)	HPV+ (12%)	I or II (27%), III or IV (73%)	PFS Status	Quantile normalised signal intensity	40	0
de Jong, 2015	GSE79493	The Netherlands	FFPE	High Pure microRNA Isolation Kit (Roche)	Illumina HiSeq 2000	Larynx (100%)	None	Male (59%), Female (42%)	67 (40-85)	NR	II (56%), III (24%), IV (21%)	Local RFS	miR counts normalised to 100,000 reads per patient	34	0
Farah, 2018	E-MTAB-6470	Australia	Fresh frozen	Trizol (Invitrogen)	SurePrint G3 Human miRNA Microarrays Release 16, 8 × 60 K (Agilent)	FOM (47%), tongue (26%), buccal mucosa (11%), gingiva (11%), hard palate (5%)	Matched adjacent tissue	Male (68%), Female (32%)	64 (26-83)	HPV+ (0%), Tobacco (59%), Alcohol (35%)	I (47%), II (6%), IV (47%)	OS, DFS	Robust Multi-array Average (RMA) without correction followed by quantile normalisation	18	36
Fukumoto, 2014	GSE51129	Japan	Fresh frozen	Trizol (Invitrogen)	miRCURY LNA microRNA Array (v7, Exiqon)	Hypopharynx (100%)	Matched adjacent hypophary-ngeal tissue	Male (73%), Female (27%)	64 (45-75)	NR	IV (100%)	NR	Background corrected and log2-transformed	11	11
Hess, 2017	E-MTAB-5198	Germany	FFPE	High Pure microRNA FFPE	GeneChip miRNA-2.0	Oropharynx (100%)	None	Male (75%),	57 (38-71)	HPV+ (15%),	IV (100%)	3-year OS status	Robust Multichip Average (RMA)	48	0

				Isolation kit (Roche)	Array (v2.0, Affymetrix)			Female (25%)		Tobacco (71%)					
Hess, 2019	E-MTAB-5793	Germany	FFPE	miRNeasy FFPE or AllPrep DNA/RNA FFPE Kit (Qiagen)	SurePrint G3 Human miRNA Microarrays, 8 × 60K (Agilent)	Hypopharynx (17%), oral cavity (36%), oropharynx (46%)	None	Male (73%), Female (27%)	59 (20-84)	HPV+ (100%)	I or II (9%), III or IV (93%)	DFS	Agilent Feature Extraction default gTotalGeneSignal	162	0
Holt, 2021	GSE144711	America	Fresh frozen	Trizol (Invitrogen)	GeneChip miRNA-1 Array (v1.0, Affymetrix)	Oral cavity (42%), oropharynx (25%), hypopharynx (8%), larynx (25%)	None	Male (76%), Female (24%)	57 (30-85)	HPV+ (11%), Alcohol (30%), Race (28%), Tobacco (72%)	I (5%), II (10%), III (16%), IV (46%)	NR	Quantile normalised RMA expression values and log2-transformed	88	0
Jung, 2012	GSE28100	America	Fresh frozen	mirVana miRNA Isolation Kit (Ambion)	Human miRNA microarray (V3, Agilent)	Tongue (88%), mouth (6%), oropharynx (6%)	Tongue	NR	61 (34-81)	HPV+ (59%)	I (12%), II (6%), III (47%), IV (29%)	OS	Variance Stabilising Normalisation	17	3
Jung, 2013	E-MTAB-1328	France	Fresh frozen	miRNeasy 96 Mini kit (Qiagen)	Illumina HiSeq 2000 (IntegraGen)	Larynopharynx (38%), mouth (11%), oropharynx (47%), tongue (5%)	None	Male (86%), Female (14%)	57 (35-77)	HPV+ (0%)	II (5%), III (18%), IV (77%)	DFS	miR counts processed by miRanalyzer 0.3 software	64	0
Lapa, 2019	GSE124678	Brazil	Fresh frozen	RNeasy kit (Qiagen) or Trizol (Invitrogen)	Unrestricted human miRNA microarray (v16.0, Agilent)	Larynx (100%)	Larynx	Male (72%), Female (28%)	62 (45-90)	Tobacco (81%), Alcohol (69%), HPV+ (6%)	I (6%), II (13%), III (25%), IV (56%)	Local-regional RFS status, Distant RFS status, OS	Intensity-dependent global normalisation (LOWESS)	32	5
Li, 2011	GSE22587	China	Fresh frozen	Trizol (Invitrogen)	Human Beta-version microRNA expression BeadChip (v1, Illumina)	Nasopharynx (100%)	Nasopharynx	Male (63%), Female (37%)	46 (24-69)	NR	II (37.5%), III (37.5%) or IV (25%)	NR	Variance-stabilising transformation and normalised with Cubic spline algorithm	8	4

Lian, 2021	GSE85608	China	Fresh frozen	Trizol (Invitrogen)	Multispecies miRNA-4 Array (v4.0, Affymetrix)	Hypopharynx (100%)	None	Male (100%)	60 (43-71)	NR	III (10%), IV (90%)	Response to chemotherapy	Quantile normalised	21	0
Liu, 2012	GSE32960	China	FFPE	Acid phenol/ chloroform extraction	MicroRNA microarray (non-commercial)	Nasopharynx (100%)	Nasopharynx	Male (75%), Female (25%)	46 (15-72)	NR	I (1%), II (18%), III (29%), IV (52%)	OS Status	Background subtraction, quantile normalisation and log2-transformed	312	18
Lyu, 2014	GSE43039	China	NR	Trizol (Invitrogen)	MicroRNA microarray (non-commercial)	Nasopharynx (100%)	Nasopharynx	Male (70%), Female (30%)	NR	NR	NR	NR	Background corrected and log2-transformed	20	20
Lyu, 2020	GSE137308	China	Fresh frozen	Trizol (Invitrogen)	Illumina HiSeq 2500 (Homo sapiens)	Larynx (100%)	Matched adjacent tissue	NR	NR	NR	III (33%), IV (67%)	NR	miRNA counts normalised to TPM (transcripts per million)	3	3
Peng, 2014b	GSE46172	America	FFPE	miRNeasy FFPE kit (Qiagen)	Human miRNA microarray (v16.0, Agilent)	Nasopharynx (100%)	Matched adjacent sinonasal mucosa or nasal polyposis	Male (75%), Female (25%)	48 (46-80)	NR	III (50%), IV (50%)	NR	Quantile normalised and log2-transformed	4	4
Saito, 2013	GSE47610	Japan	FFPE	mirVana miRNA Isolation Kit (Ambion)	Human miRNA microarray (v2, Agilent)	Larynx (100%)	Matched adjacent laryngeal tissue	NR	NR	NR	I (67%), II (33%)	NR	Normalised signal intensity (gTotalGeneSignal)	3	2
Severino, 2013	GSE31277	Brazil	Fresh frozen	mirVana miRNA Isolation Kit (Ambion)	miRNA microarray (v1.0, Illumina)	Tongue (40%), mouth (60%)	Matched adjacent (surgical margin)	Male (93%), Female (7%)	56 (46-82)	NR	I (7%), II (27%), III (7%), IV (60%)	NR	Quantile normalized	15	15
Shi, 2015	GSE32115	China	Fresh frozen	Trizol (Invitrogen)	Custom Homo sapiens miRNA array (v9.2, Exiqon)	Oral cavity (100%)	Matched adjacent mucosal tissue	NR	NR	NR	NR	NR	Background subtraction, median normalized	2	2
Shiah, 2014	GSE45238	Taiwan	Fresh frozen	miRNeasy Mini kit (Qiagen)	Human MicroRNA Expression	Oral cavity (100%)	Matched adjacent	Male (100%)	49 (36-72)	NR	I (3%), II (35%), III (25%),	NR	Quantile normalized	40	40

					BeadChips (v2, Illumina)		mucosal tissue				IV (37%)				
Stansfield, 2016	GSE34496	America	Fresh frozen	Trizol (Invitrogen)	Multispecies miRNA-1 Array (v1.0, Affymetrix)	Oral cavity (23%), oropharynx (38%), larynx (30%), hypopharynx (9%)	Uvulopalat-opharyngo-plasty tissue	Male (73%), Female (27%)	58 (45-80)	Tobacco (61%), Alcohol (57%), HPV+ (30%)	I (11%), II (5%), III (11%), IV (73%)	NR	Robust Multichip Average	44	25
TCGA	HNSC	America	Fresh frozen	miRVana (allprep DNA) RNA hybrid protocol	Illumina GA IIx, Illumina Hiseq 2000 (Homo sapiens)	Hypopharynx (2%), larynx (22%), oral cavity (61%), oropharynx (15%)	Matched adjacent tissue	Male (73%), Female (27%)	61 (20-90)	Tobacco (75%), Alcohol (68%), HPV+ (19%)	I (4%), II (19%), III (21%), IV (56%)	OS, DSS, DFS, PFS	Log2(RPM+1)	514	40
Wu, 2020	GSE133632	China	Fresh frozen	Trizol (Invitrogen)	Illumina HiSeq 2000 (Homo sapiens)	Larynx (100%)	Matched adjacent tissue	Male (93%), Female (7%)	NR	Tobacco (47%), Alcohol (28%)	I (18%), II (9%), III (13%), IV (10%), NA (50%)	NR	miRNA counts normalised to TPM (transcripts per million)	57	57
Yang, 2018	GSE62819	China	Fresh frozen	mirVana RNA Isolation Kit (Ambion)	Multispecies miRNA-3 Array (v3.0, Affymetrix)	Larynx (100%)	Matched adjacent laryngeal tissue	Male (100%)	56 (53-74)	NR	II (20%), III (20%), IV (60%)	NR	Log2-transformed GeneChip-Robust Multichip Average	5	5
Yoon, 2014	GSE52633	America	FFPE	RNeasy FFPE kits (Qiagen)	Illumina HiSeq 2500 (Homo sapiens)	Oral mucosa (100%)	None	Male (50%), Female (50%)	NR	Tobacco (50%), Alcohol (25%)	I (65%), II (35%)	5-year OS status	Miraligner miR counts	20	0
Yoon, 2020	GSE107830	America	FFPE	RNeasy FFPE kits (Qiagen)	Illumina NextSeq 500 (Homo sapiens)	Oral mucosa (100%)	None	NR	NR	NR	NR	Death/ recurrence status	log2(counts per million)	91	0
Zhang, 2016	GSE81821	China	Fresh frozen	Trizol (Invitrogen)	Multispecies miRNA-2 Array (v2.0, Affymetrix)	Tongue (100%)	None	NR	NR	NR	NR	Metastasis status	Quantile normalised	10	0
Zhao, 2019	GSE116994	China	Fresh frozen	Trizol (Invitrogen)	Multispecies miRNA-4	Larynx (100%)	Matched adjacent	NR	NR	NR	III (40%), IV (60%)	NR	Robust Multichip Average	5	5

					Array (v4.0, Affymetrix)		laryngeal tissue									
Zheng, 2018	GSE107445	China	Fresh	Trizol (Invitrogen)	Illumina Genome Analyzer IIx (Homo sapiens)	Oral cavity (100%)	Matched adjacent tissue	NR	NR	NR	NR	NR		miRNA counts normalised to TPM (transcripts per million)	4	4
Zhuang, 2020	GSE124566	China	Fresh frozen	mirVana miRNA Isolation Kit (Ambion)	Unrestricted Human miRNA microarray (v19.0, Agilent)	Tongue (100%)	Matched adjacent tongue tissue	Male (70%), Female (30%)	50 (32-64)	NR	II (30%), III (20%), IV (50%)	NR		Quantile normalised	10	10

[1] NR, Not Reported; FOM, Floor of mouth; OS, Overall Survival; DFS, Disease-free Survival; RFS, Relapse-free Survival; PFS, Progression-free Survival; DSS, Disease-specific Survival.

Table S5. Summary of meta-analysis results: all studies.

Subgroup meta-analysis summary | Number of studies = 24

Random-effects model

Method: REML

Groups: site, method of normalisation

Group	No. of studies	Hedges's g	95% CI		p
Site of tumour					
Pharynx	6	0.227	-0.220	0.675	0.320
Oral cavity	9	1.48	1.06	1.90	<0.001
Larynx	6	0.808	0.491	1.12	<0.001
Mixed Head & Neck	3	0.990	0.132	1.85	0.024
Method of normalisation					
Variance-stabilising transformation	4	0.683	-0.144	1.51	0.106
Background corrected	3	0.083	-0.52	0.685	0.787
Quantile normalised	5	1.97	1.63	2.32	<0.001
miR counts normalised	3	0.943	0.586	1.30	<0.001
Log2-transformed relative to normal	3	0.96	0.668	1.25	<0.001
Robust Multichip Average	3	0.636	0.063	1.21	0.030
Other	3	0.473	-0.192	1.14	0.163
Overall: theta	24	0.917	0.602	1.23	<0.001

Heterogeneity summary

Group	d.f.	Q	p	tau2	% I2	H2
Site of tumour						
Pharynx	5	12.2	0.032	0.160	56.6	2.30
Oral cavity	8	16.0	0.043	0.194	50.4	2.02
Larynx	5	3.77	0.584	0.002	1.05	1.01
Mixed Head & Neck	2	8.92	0.012	0.440	84.8	6.60
Method of normalisation						
Variance-stabilising transformation	3	8.35	0.039	0.465	68.0	3.13
Background corrected	2	5.26	0.072	0.179	64.1	2.78
Quantile normalised	4	2.34	0.673	<0.001	<0.001	1.00
miR counts normalised	2	1.19	0.551	<0.001	<0.001	1.00
Log2-transformed relative to normal	2	0.500	0.780	<0.001	<0.001	1.00
Robust Multichip Average	2	2.71	0.258	0.087	30.5	1.44
Other	2	1.67	0.435	0.002	0.430	1.00
Overall	23	96.7	<0.001	0.405	76.0	4.17

Tests of group differences	d.f.	Q	p
Site of tumour	3	16.3	0.001
Method of normalisation	6	43.9	<0.001

Table S6. Meta-regression results examining the impact of tumour site on effect size, highlighting oral cavity as a significant contributor to increased expression differences.

Site	Coefficient	Standard Error	z	p	95% CI	
Pharynx	1 (ref.)					
**Oral cavity**	**1.26**	**0.307**	**4.10**	**<0.001**	**0.656**	**1.86**
Larynx	0.507	0.350	1.45	0.147	-0.178	1.19
Mixed sites	0.673	0.376	1.79	0.074	-0.065	1.41
Constant	0.228	0.229	0.990	0.320	-0.221	0.678
Test of residual homogeneity: Q residual= 40.9, p = 0.0039						

Table S7. Meta-regression results examining the impact of normalisation method on effect size, highlighting quantile normalisation as a significant contributor to increased expression differences.

Site	Coefficient	S.E.	z	p	95% CI	
Variance-stabilising transformation	1 (ref.)					
Background corrected	-0.495	0.323	-1.54	0.125	-1.13	0.137
**Quantile normalised**	**1.44**	**0.315**	**4.58**	**<0.001**	**0.826**	**2.06**
miR counts normalised	0.441	0.346	1.27	0.203	-0.237	1.12
Log2-transformed relative to normal	0.407	0.322	1.27	0.206	-0.223	1.04
Robust Multichip Average	0.080	0.351	0.23	0.820	-0.609	0.769
Other	-0.023	0.434	-0.05	0.958	-0.873	0.827
Constant	0.515	0.239	0.032	0.032	0.045	0.984
Test of residual homogeneity: Q residual= 22.0, p = 0.184						

Table S8. Patient Characteristics of the Perkins Cancer Biobank Cohort.

PCB#	Sex	Age	Site	Stage	TNM	Alcohol Use	Tobacco Use
312	F	60	tongue, dorsal surface	IVA	T3N2aMX	Yes	No
330	M	68	tongue	II	T2N0M0	Yes	Yes
366	M	77	floor of mouth	III	T3N0MX	Yes	Yes
404	M	65	floor of mouth	IV	T4N1M0	Yes	Yes
424	F	61	tongue	II	T2N0M0	No	No
436	M	69	tongue, right lateral	IV	T4aN1MX	Yes	Yes
443	M	77	floor of mouth, anterior	IVA	T4N2M0	No	No
451	F	80	floor of mouth, lateral	I	T1N0M0	No	No
459	F	61	tongue	III	T4N2bMX	NR	Yes
464	F	54	floor of mouth, lateral	I	T1N0M0	No	No
466	F	79	floor of mouth	IVA	T4aN0MX	No	No
471	M	74	sinonasal right maxilla	III	T3N0M0	No	No
474	M	81	floor of mouth, lateral	IVB	T3N3bM0	Yes	Yes
481	M	65	tongue	IVA	T3N2M0	Yes	Yes
489	M	79	tongue, left lateral	IVA	T4N0M0	Yes	Yes
494	M	61	floor of mouth	IVA	T2N3bMX	NR	Yes

NR, Not reported

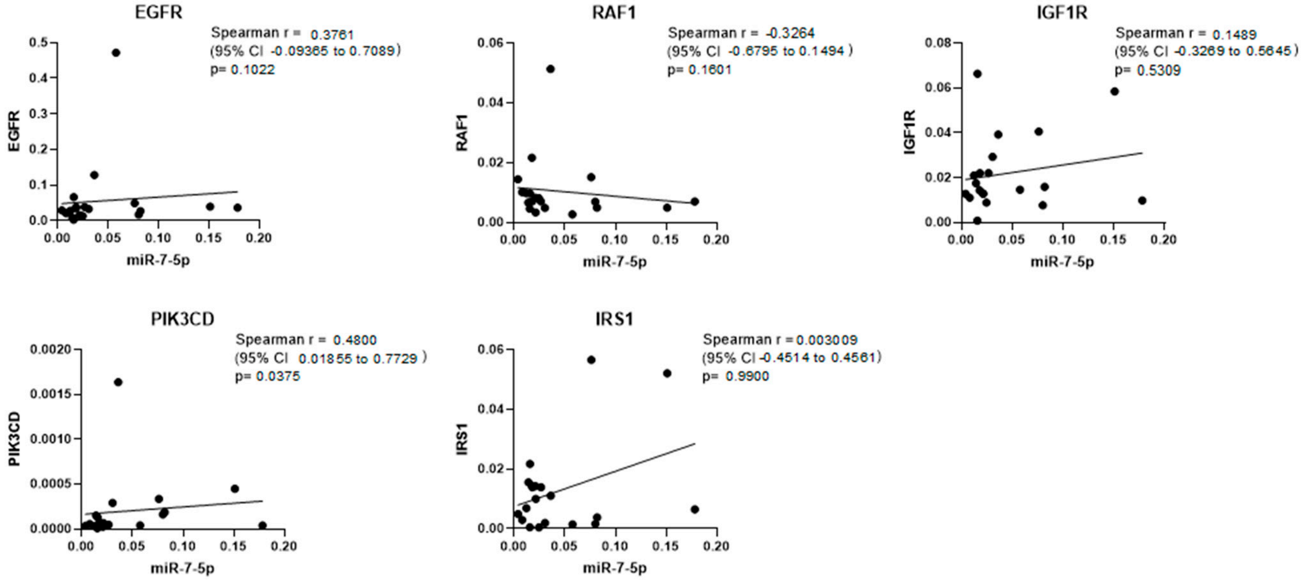

Figure S1. Correlation of miR-7-5p expression with target genes in patient samples PCB cohort (n=16 tumours and n=4 normal samples). miR-7-5p and hub gene expression from head and neck cancer patients was quantified by RT-qPCR. Data are shown as $2^{-\Delta Ct}$ values, normalised to U6 and GAPDH as endogenous controls. Each sample was reverse transcribed once and qPCR run with >=3 technical replicates. Statistical analysis: Spearman r correlation coefficient, p <0.05 considered significant.

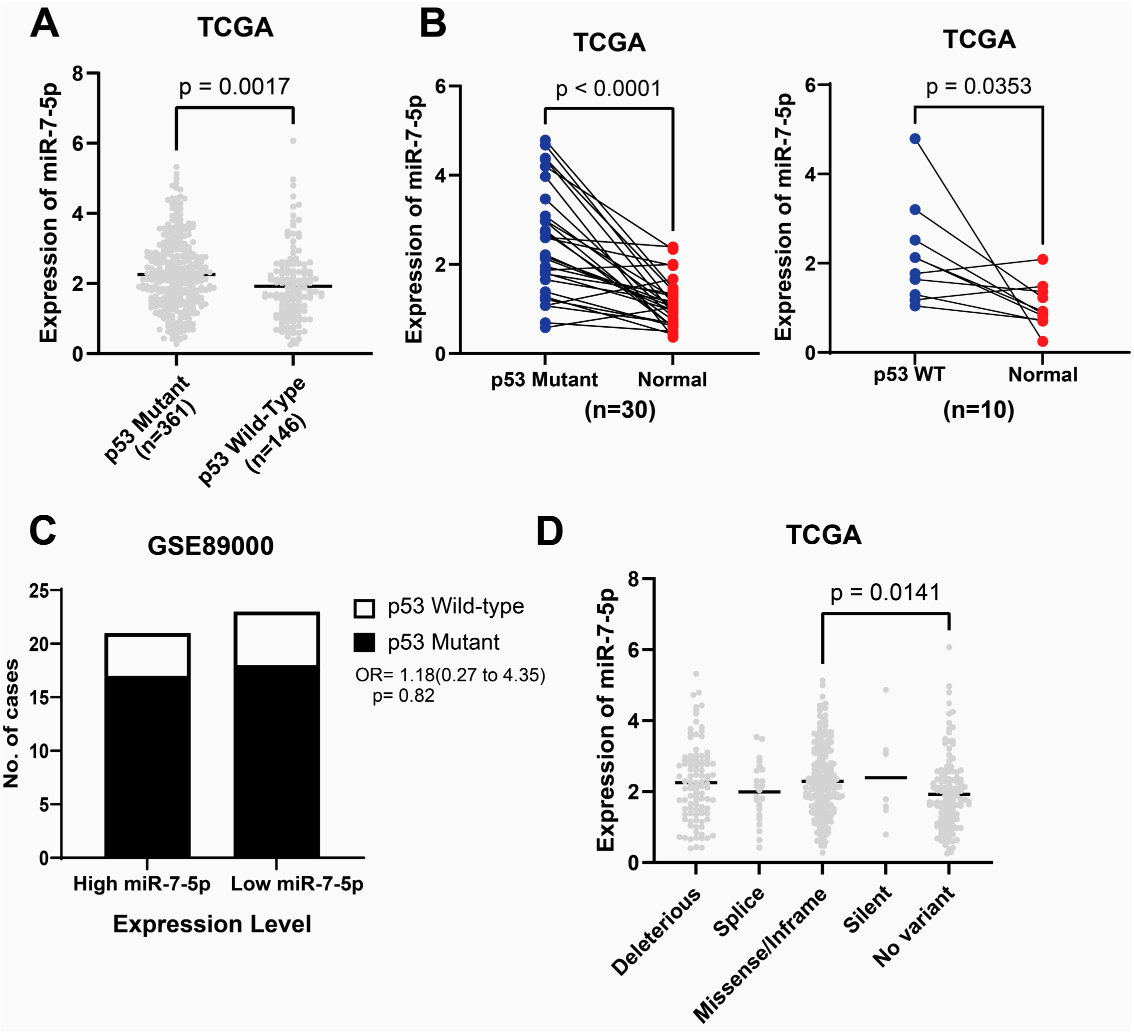

Figure S2. Association of miR-7-5p expression with p53 status in HNSCC. (A) Expression levels of miR-7-5p in HNSCC from the TCGA cohort separated by TP53 status (mutant versus wild-type). Unpaired, 2-tailed, *t*-test, $p < 0.05$ considered significant. (B) Expression levels of miR-7-5p in HNSCC TCGA cohort separated by type pf TP53 mutation. One-way ANOVA, Tukey's post-hoc comparisons, $p < 0.05$. (C) Expression levels of miR-7-5p in HNSCC from the TCGA cohort with wild-type TP53 and matched normal tissue (n=10). Paired *t*-test, 2-tailed, $p < 0.05$ considered significant. (D) miR-7-5p (miR-7) expression levels in tumours from GEO89000 dataset grouped by p53 status. OR, Odds Ratio calculated from Chi-square test.

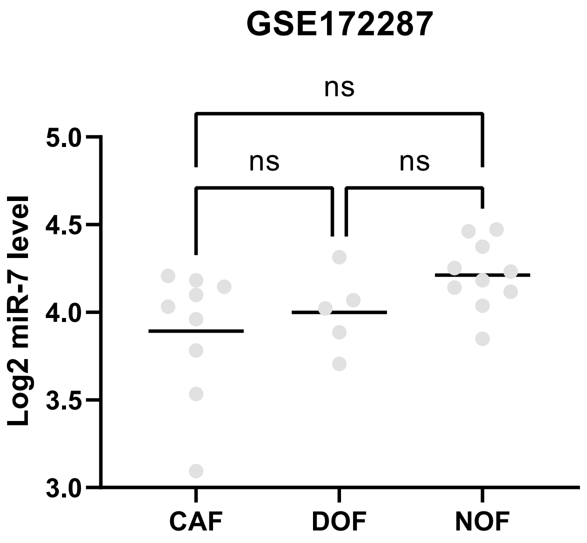

Figure S3. Public array data (GSE172287) showing miR-7-5p (miR-7) expression levels in oral cancer-associated fibroblasts (CAFs), dysplastic oral fibroblasts (DOFs), and normal oral fibroblasts (NOFs). Samples were from primary OSCC lesions (n=9), adjacent normal regions (n=5) and healthy individuals (n=9), respectively. Ns, not significant determined by One-way ANOVA with Tukey's multiple comparisons test.

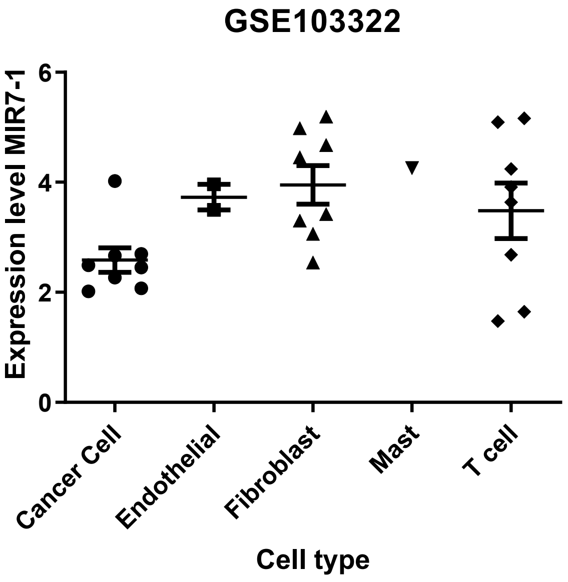

Figure S4. Expression of MIR7-1 precursor stemloop structure in various cell types from single cell RNA-sequencing of human oral cavity tumours (n=18). Dataset includes 5902 cells in total (including 2215 cancer cells, 260 endothelial cells, 1440 fibroblasts, 120 mast cells, 1237 T cells), and there are 29 cells with MIR7-1 expression. Not significant using Fisher's test for association between cell types and expression levels.

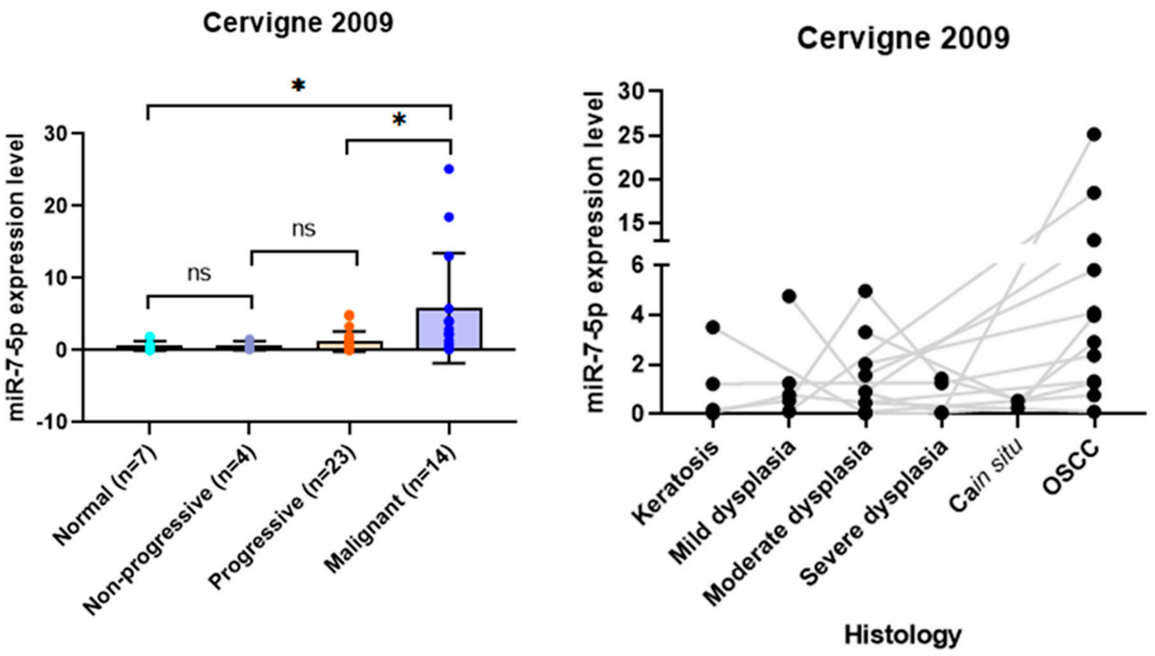

Figure S5. Expression of miR-7-5p in normal, dysplastic and cancer oral tissues from Cervigne *et al* [1] taken from 43 sequential progressive samples from 12 patients and four non-progressive leukoplakias from four different patients. One-way ANOVA with Tukey's post-hoc multiple comparisons, $p < 0.05$ considered significant.

Reference

1. Cervigne, N.K.; Reis, P.P.; Machado, J.; Sadikovic, B.; Bradley, G.; Galloni, N.N.; Pintilie, M.; Jurisica, I.; Perez-Ordonez, B.; Gilbert, R.; et al. Identification of a microRNA signature associated with progression of leukoplakia to oral carcinoma. *Hum. Mol. Genet.* **2009**, *18*, 4818–4829. https://doi.org/10.1093/hmg/ddp446.
